# Women’s and men’s reports of past-year prevalence of intimate partner violence and rape and women’s risk factors for intimate partner violence: A multicountry cross-sectional study in Asia and the Pacific

**DOI:** 10.1371/journal.pmed.1002381

**Published:** 2017-09-05

**Authors:** Rachel Jewkes, Emma Fulu, Ruchira Tabassam Naved, Esnat Chirwa, Kristin Dunkle, Regine Haardörfer, Claudia Garcia-Moreno, on behalf of the UN Multi-country Study on Men and Violence Study Team

**Affiliations:** 1 Gender & Health Research Unit, Medical Research Council and School of Public Health, University of the Witwatersrand, Pretoria, South Africa; 2 The Equality Institute, Melbourne, Australia; 3 International Centre for Diarrhoeal Disease Research, Dhaka, Bangladesh; 4 Emory University, Atlanta, Georgia, United States of America; 5 Department of Reproductive Health and Research, World Health Organization, Geneva, Switzerland; Massachusetts General Hospital, UNITED STATES

## Abstract

**Background:**

Understanding the past-year prevalence of male-perpetrated intimate partner violence (IPV) and risk factors is essential for building evidence-based prevention and monitoring progress to Sustainable Development Goal (SDG) 5.2, but so far, population-based research on this remains very limited. The objective of this study is to compare the population prevalence rates of past-year male-perpetrated IPV and nonpartner rape from women’s and men’s reports across 4 countries in Asia and the Pacific. A further objective is to describe the risk factors associated with women’s experience of past-year physical or sexual IPV from women’s reports and factors driving women’s past-year experience of partner violence.

**Methods and findings:**

This paper presents findings from the United Nations Multi-country Study on Men and Violence in Asia and the Pacific. In the course of this study, in population-based cross-sectional surveys, 5,206 men and 3,106 women aged 18–49 years were interviewed from 4 countries: Cambodia, China, Papua New Guinea (PNG), and Sri Lanka. To measure risk factors, we use logistic regression and structural equation modelling to show pathways and mediators. The analysis was not based on a written plan, and following a reviewer’s comments, some material was moved to supplementary files and the regression was performed without variable elimination. Men reported more lifetime perpetration of IPV (physical or sexual IPV range 32.5%–80%) than women did experience (physical or sexual IPV range 27.5%–67.4%), but women’s reports of past-year experience (physical or sexual IPV range 8.2%–32.1%) were not very clearly different from men’s (physical or sexual IPV range 10.1%–34.0%). Women reported much more emotional/economic abuse (past-year ranges 1.4%–5.7% for men and 4.1%–27.7% for women). Reports of nonpartner rape were similar for men (range 0.8%–1.9% in the past year) and women (range 0.4%–2.3% in past year), except in Bougainville, where they were higher for men (11.7% versus 5.7%). The risk factor modelling shows 4 groups of variables to be important in experience of past-year sexual and/or physical IPV: (1) poverty, (2) all childhood trauma, (3) quarrelling and women’s limited control in relationships, and (4) partner factors (substance abuse, unemployment, and infidelity). The population attributable fraction (PAF) was largest for quarrelling often, but the second greatest PAF was for the group related to exposure to violence in childhood. The relationship control variable group had the third highest PAF, followed by other partner factors. Currently married women were also more at risk. In the structural model, a resilience pathway showed less poverty, higher education, and more gender-equitable ideas were connected and conveyed protection from IPV. These are all amenable risk factors. This research was cross-sectional, so we cannot be sure of the temporal sequence of exposure, but the outcome being a past-year measure to some extent mitigates this problem.

**Conclusions:**

Past-year IPV indicators based on women’s reported experience that were developed to track SDG 5 are probably reasonably reliable but will not always give the same prevalence as may be reported by men. Report validity requires further research. Interviews with men to track past-year nonpartner rape perpetration are feasible and important. The findings suggest a range of factors are associated with past-year physical and/or sexual IPV exposure; of particular interest is the resilience pathway suggested by the structural model, which is highly amenable to intervention and explains why combining economic empowerment of women and gender empowerment/relationship skills training has been successful. This study provides additional rationale for scaling up violence prevention interventions that combine economic and gender empowerment/relationship skills building of women, as well as the value of investing in girls’ education with a view to long-term violence reduction.

## Abstract 2

In a cross-sectional analysis of survey data from Cambodia, China, Papua New Guinea, and Sri Lanka, Rachel Jewkes and colleagues compare women's and men's reports of past-year prevalence of intimate partner violence and rape.

## Author summary

**Why was the study done?:**

- Understanding the past-year prevalence of physical and or sexual intimate partner violence (IPV) and risk factors is essential for building evidence-based prevention.
- Previous studies have not compared men’s and women’s past-year prevalence reports and have been limited by a predominant focus on risk factors for lifetime exposure to IPV.
- Monitoring SDG 5.2 and building evidence-based prevention require a relative understanding of the measures of past-year prevalence and the drivers of this violence.

**What did the authors do and find?:**

- We use data from 4 countries of the UN Multi-country Study on Men and Violence in Asia and the Pacific to compare the population prevalence rates of past-year IPV and nonpartner rape from women’s and men’s reports and present an analysis of drivers of women’s experience of past-year physical or sexual IPV.
- Women’s reports of past-year male-perpetrated IPV were similar to those from men.
- Four groups of variables are important drivers of IPV: poverty, all childhood trauma, quarrelling and women’s limited control in the relationship, and partner factors (substance abuse, unemployment, and infidelity).

**What do these findings mean?:**

- Past-year IPV indicators based on women’s reported experience that were developed to track SDG 5 are probably reasonably reliable.
- Women appear to gain resilience to violence through combined economic power and understanding gender empowerment/relationship skills, as well as education; this is an important foundation for intervention.
- Further research is needed on the validity of men’s and women’s reports of IPV, which could not be determined from these data.

## Introduction

In 2015, eliminating all forms of violence against women and girls (VAWG) was adopted as a target for the Sustainable Development Goal (SDG) 5 on gender equality and empowerment of women. To achieve this, we must develop and roll out effective measures to prevent male-perpetrated violence and show their effect. The indicators of progress towards this target are not finalized but will be a measure of women’s experience of intimate partner violence (IPV) and of nonpartner sexual violence in the past 12 months. According to most recent estimates, 30% of women aged 15 years and over have experienced male-perpetrated physical and/or sexual IPV, and 7% nonpartner sexual violence, in their lifetime [1,2].

In low- and middle-income countries, the World Health Organization instrument that was developed for its Multi-country Study on Women’s Health and Domestic Violence against Women is generally seen as the gold standard measure for women. Parallel research with men has developed a methodology for measuring perpetration, but the 2 measures of violence in heterosexual relationships have not been compared. Given that widely used indicators will most likely focus on reports of just 1 gender for reasons of resource constraints, it is important that there be an understanding of the comparability of men’s and women’s reports. Without this, we have uncertainty about the validity of women’s reports of experiences of IPV and nonpartner sexual violence. There is particular concern that sexual violence may be under-reported by women because rape is highly stigmatized, which may result in minimization of events, but it is also possible men might under-report perpetration of violence so as not to incriminate themselves [3,4].

Prevention of VAWG needs to be built on evidence of drivers among women currently at risk (as well as those of perpetration). There is a reasonably large amount of literature on risk factors for experience of IPV (for example, summarized in the World Health Organization’s 2010 review [5]), but major limitations include a focus on lifetime exposure (rather than past year) and overadjustment of models for (nonamenable) at-risk groups rather than focusing on risk factors. In the case of the former, this means that the outcome modelled is not exactly the ‘problem’ for which interventions are required (which is current, or future, violence). In the case of the latter, the analyses focus largely on who is at risk rather than understanding factors driving risk. The literature is also mostly focused on a single country and is cross-sectional [6], and given the variability in the variables measured and the modelling approaches used, this often constrains the ability to compare across countries and global regions. Prevention science is better informed by looking at risk factors amenable to intervention and linked to past-year experience of IPV, which are likely to differ from factors associated with lifetime experience of IPV.

The UN Multi-country Study on Men and Violence was designed to address many of the gaps in previous data sources [7]. It has a large multicountry dataset with women’s reports of IPV, collected using gold standard exposure measures, and also includes standard measures of the most important currently recognised drivers of violence as well as some hypothesised ones. We present the population prevalence of women’s experiences of past-year IPV and nonpartner rape and compare it to men’s reported perpetration across 4 countries in Asia and the Pacific, and we present risk factors associated with women’s experience of past-year physical or sexual IPV (risk factors for men’s perpetration in this dataset have been presented previously [8,9]). We present structural models to show pathways and mediators.

## Methods

Ethical approval was provided by the Medical Research Council of South Africa; the College of Humanities, Beijing Forestry University; National Ethics Committee for Health Research of Cambodia; and the Faculty of Medicine at the University of Colombo, Sri Lanka.

The survey was developed by Partners for Prevention in collaboration with the Medical Research Council of South Africa and the country research teams. Research was conducted in 2011–2012. Of the 6 country surveys, only 4 had male and female interviews: China, Cambodia, Bougainville in Papua New Guinea, and Sri Lanka. The present study is intended to contrast women's reported experience of IPV and nonpartner rape with men's reported perpetration of IPV and nonpartner rape; therefore, our analysis focuses on these 4 surveys. The sample from Cambodia and the sample from Papua New Guinea were representative, respectively, of Cambodia and the island of Bougainville. The Chinese site was a county with a town and rural area, and in Sri Lanka, Colombo and 3 contrasting districts were surveyed. Further details of the research can be found elsewhere [7,10].

In each setting, we selected census enumeration areas, with a probability proportionate to size, and systematically selected households within these areas. In households, we invited a man or woman (depending on the cluster) aged 18–49 years (where necessary, randomly selected) for interview, with a trained sex-matched interviewer. Most interviews were face to face, but for men, answers to most sensitive questions were self-completed on audio-enhanced personal digital assistants (APDAs). In China, a household list of individuals in each cluster by age and sex was available and used for sampling within selected clusters, and the entire questionnaire was self-completed. Full details of the methods, sampling, and response rates are presented elsewhere [10]. We conducted surveys with women on their health and experiences of violence in 4 sites (Cambodia, China, Bougainville, and Sri Lanka). We sampled men and women in separate clusters. We conducted interviews with 3,106 women (between 477–1,103 per country) and 5,206 men (between 849–1,777 per country across the 4 analysed here). The proportion of enumerated and eligible women interviewed per site was between 92.7% (in Cambodia) and 73.9% (in Sri Lanka). For men, it ranged between 97.3% (in Cambodia) and 58.7% (in Sri Lanka; for details [7]). Measures used in the questionnaire are presented in Table 1. We followed ethical and safety guidelines for research on violence against women [11,12]. The interviewees received an information sheet and provided written consent.

**Table 1:** Measures.

Construct	Indicator	Definition
**Violence against women**		
	Physical IPV	The score was based on 5 behaviourally specific items asked about the actions of a current or former partner in the last year and 5 before the past year: was slapped or had something thrown at her that could hurt her; was pushed or shoved; was hit with a fist or something else that could hurt her; was kicked, dragged, or beaten up; or current/former partner threatened to use or actually used a gun, knife, or other weapon against her. These items were developed from Garcia-Moreno et al. (2005), and each had ‘never’, ‘once’, ‘a few times’, or ‘often’ response options.
	Sexual IPV (men)	2 items: He forced partner to have sex when she did not want to; he had sex with partner when he knew she did not want to, but he believed she should agree because she was his wife/partner.
	Sexual IPV (women)	2 items: He physically forced her to have sex when she did not want to; she had sex with a current/former partner when she did not want to because she was afraid of what he might do to her.
	Emotional IPV	5 items: Current/former partner ever insulted or deliberately made her feel bad about herself; belittled or humiliated her in front of other people; did things to scare or intimidate her on purpose (e.g., by yelling or smashing things); threatened to hurt her; or hurt people she cares about as way of hurting her or damaged things that were important to her.
	Economic IPV	2 items: Current/former partner prohibited her from getting a job, going to work, trading, or earning money; current/former partner took her earnings against her will.
Physical IPV only		Respondent experienced/perpetrated at least 1 act of physical IPV or experienced/perpetrated at least 1 act of physical IPV and emotional/economic abuse but did not experience/perpetrate any acts of sexual IPV.
Sexual IPV only		Respondent experienced/perpetrated at least 1 act of sexual IPV or experienced/perpetrated at least 1 act of sexual IPV or emotional/economic abuse but did not experience/perpetrate any acts of physical IPV.
Both sexual and physical IPV		Experienced/perpetrated at least 1 act of sexual IPV and at least 1 act of physical IPV, with or without emotional/economic abuse.
Multiple emotional/economic only		Respondent experienced/perpetrated more than 1 act of emotional or economic abuse, or 1 act several times, from/on intimate partner but never experienced/perpetrated sexual or physical IPV.
	Nonpartner rape (men)	2 items: asked about having forced a woman who was not his wife or girlfriend at the time to have sex; having had sex with a woman who was too drunk or drugged to indicate whether she consented. 2 further items were asked using the same structure but with the formulation ‘with other men’.
	Nonpartner rape (women)	3 items: forced or persuaded to have sex against her will by a man who was not her husband or boyfriend; forced to have sex with a man who was not a husband or boyfriend when too drunk or drugged to refuse; or forced or persuaded to have sex against her will with more than 1 man at the same time.
	Hunger	The respondent was asked the following: ‘Would you say that the people in your home often, sometimes, seldom, or never go without food?’
	Resource mobilisation	The respondent was asked the following: ‘If a person became ill in your home and [about US$10] was needed for treatment or medicines, would you say it would be very easy, easy, quite difficult, or very difficult to find the money?’
Wealth score	(Exogenous in structural model)	Sum of the hunger and emergency resource mobilisation variables. 8-point scale.
Childhood trauma	Childhood emotional abuse or neglect	Based on a modified version of the Childhood Trauma Questionnaire: Before age 18 years, the respondent had at least 1 of the following experiences sometimes, often, or very often: lived in different households at different times; was told she was lazy or stupid or weak by someone in her family; was insulted or humiliated by someone in her family in front of other people; both of her parents were too drunk or drugged to take care of her; or spent time outside the home and none of the adults at home knew where she was.
	Physical abuse	Before age 18 years, the respondent had at least 1 of the following experiences sometimes, often, or very often: was beaten at home with a belt, stick, whip, or something else that was hard; was beaten so hard at home that it left a mark or bruise.
	Sexual abuse	Before age 18 years, the respondent had at least 1 of the following experiences sometimes, often, or very often: someone touched her buttocks or genitals or made her touch them when she did not want to; or she had sex with someone because she was threatened, frightened, or forced.
Childhood trauma overall score		Childhood trauma score derived from the sum of the about 3 subscales (Cronbach’s Alpha was 0.74).
	Witnessing abuse of mother	Before age 18, the respondent saw or heard her mother being beaten by her husband or boyfriend
	Partner drug use	Single item asking about how often the partner uses drugs.
	Partner alcohol use	Single item asking about how often the partner drinks.
	Gender-equitable attitudes	A scale was created from 8 items scored on a 4-point scale from strongly agree to strongly disagree: ‘A woman’s most important role is to take care of her home and cook for her family’; ‘Men need more sex than women do’; ‘I would be outraged if my husband asked me to use a condom’; ‘There are times when a woman deserves to be beaten’; ‘It is a woman’s responsibility to avoid getting pregnant’; ‘A woman should tolerate violence in order to keep her family together’; ‘If someone insults a man I would expect him to defend his reputation with force, if he needs to’; and ‘To be a man, you need to be tough.’
	Partner faithfulness	The respondent was asked the following: ‘How likely do you think it is that your current/most recent husband/partner is having sex with someone else? Would you say he definitely is, probably is, probably is not, or definitely is not?’
	Controlling behaviour	Partner is moderately or highly controlling over female partner compared with least controlling, based on 8 items scored on a 4-point scale from strongly agree to strongly disagree: ‘When I want sex, I expect my partner to agree’; ‘If my partner asked me to use a condom, I would get angry’; ‘I won’t let my partner wear certain things’; ‘I have more to say than she does about important decisions that affect us’; ‘I tell my partner who she can spend time with’; ‘When my partner wears things to make her look beautiful, I think she may be trying to attract other men’; ‘I want to know where my partner is all of the time’; and ‘I like to let her know she isn’t the only partner I could have.’
	Quarrelling	Respondent quarrels with current or most recent intimate partner rarely, sometimes, or often.

IPV, intimate partner violence.

### Data analysis

The data analysis was largely planned at the point of commencement of the work on the paper. Authors EF and RJ were involved in the research from its inception and had planned the questionnaire so that it would be possible to undertake an analysis of prevalence of violence and risk factors. They ensured as much as possible that the main variables previously described in the literature [5] were included in the dataset. We planned the analysis to test the relationships between the independent variables and the outcomes. This study is reported as per the STROBE guidelines (S1 STROBE Checklist).

We combined the datasets and analysed the data using Stata, version 13. All procedures took into account the multistage structure of the dataset, with stratification by site within a country and enumeration areas as clusters. The sample was self-weighting. Women’s experiences of violence and male partner violence perpetration, as well as the independent variables, were summarized as percentages (or means), with 95% confidence limits calculated using standard methods (Taylor linearization).

We categorised the type of violence exposure according to the most severe type experienced, where greatest severity was considered as exposure to physical and/or sexual IPV, as this is the category that has been the basis of most health consequences research [1] and is consistent with the paper on male risk factors for IPV published from the same dataset [10]. It is currently common practice in the field not to model a combined variable with sexual and physical IPV and economic and emotional abuse, although this has been sometimes done [13]. This is because the field’s understanding of the latter is at a much earlier stage, with limited agreement on how to measure it, how to prevent it, and the implications (of emotional abuse alone) for health and development outcomes. It is important for the field that the issue is not ignored, hence its inclusion here, but we do not feel that the field is quite ready for it to be meaningfully pooled with sexual and physical violence for risk factor modelling and interventions. This approach has been followed by other authors, for example, Mahenge et al. [14].

The multiple emotional/economic abuse category consisted of women who had experienced more than 1 act of economic or emotional abuse but never experienced sexual or physical abuse. All ever-partnered women and men were classified into 5 violence exposure categories: none, emotional/economic without sexual or physical (henceforth referred to as ‘emotional/economic’), sexual without physical and with or without emotional/economic (henceforth referred to as ‘sexual’), physical without sexual and with or without emotional/economic (henceforth referred to as ‘physical’), or sexual and physical with or without emotional/economic (henceforth referred to as ‘physical/sexual’).

We also evaluated the relationship between the outcome (IPV) and nonresponse (missing data) in putative risk factors. No association was found between a woman’s IPV status and her nonresponse to any of the possible risk factors. However, to increase the sample of women with responses to scale measurements (e.g., gender attitudes and relationship control), women with partial responses to scale items were also included. Three methods for imputing for missing data were initially compared. These involved imputing for missing scale items using either (1) a woman’s responses to other items in the scale (individual respondent mean) or (2) the average for each item adjusted for IPV status, or (3) the average of the overall score adjusted for IPV status. There were no significant differences in the 3 methods for both gender attitudes and relationship control scores. We used ‘the average of the overall score (adjusted for IPV status)’ to impute for missing scores.

The exercise of testing variables and building model drew on current theories about risk factors and drivers of violence against women. The selection of variables as putative risk factors was informed by the state of knowledge in the field. Drawing on a life-course modified ecological model of violence risk [15], we conceptualized possible risk factors as (1) structural, (2) those pertaining to the women (including stemming from her childhood), (3) those pertaining to her partner, and (4) those pertaining to their relationship. We further were informed in our thinking by research on masculinities that views a range of male behaviours as indicator variables for hegemonic masculinity [16]. The connections between hegemonic masculinity and violence against women have been extensively theorized. In building the structural equation model, we drew on our extensive knowledge base on gender-based violence. It is well recognized that IPV is strongly associated with poverty and that poverty increases the likelihood of experience of adversity in childhood and influences access to education [9,17–19]. Research with men has shown that childhood trauma exposure influences ideas about gender equity, which is why we hypothesized this direction of effect for women [19]. Further research has shown that women’s ideas about gender influence partner selection, as does exposure to childhood trauma [20].

To show associations between independent variables that were putative risk factors, we first conducted a bivariable analysis with a (by type) lifetime IPV exposure measure and a multinomial regression with no physical, sexual, or severe economic/financial violence as the comparison group. A maximum likelihood multinomial logit model, which adjusted for the survey design, was used to compare factors associated with different types of IPV experienced with the no-violence reference category. We initially fitted bivariable models and then included all factors that were significantly associated with IPV experience in the bivariate models into an overall model, which was adjusted for the country and age group of the woman.

We examined factors associated with past-year experience of IPV considering the same independent variables, but with a past-year exposure to any physical and/or sexual IPV as the outcome, due to sample size considerations, we did not perform multinomial modelling. We sought to model 19 covariates in the logistic regression model, which, according to generally accepted rules of thumb [21], would require a total of 190 events. Because the category ‘physical and sexual IPV’ contained only 124 events, having this as an outcome in a multinomial model could have resulted in overfitting. We therefore decided to fit a regression model specifying the combined outcome of ‘any exposure to physical and/or sexual IPV’. Since this combined outcome contained 2,765 x 16.7% = 461 events, this decision allowed us to proceed with less concern about overfitting.

Multivariable logistic regression was used to determine risk factors associated with past-year physical and/or sexual IPV experience in women, with those not experiencing this as the reference group. To enable the use of a variable on frequency of quarrelling, which was not measured in Cambodia, a dummy level for Cambodia was created for the quarrelling variable for use in the logistic regression model. All variables were included in the multivariate analysis. We focus the discussion on variables with *P* < or = 0.05 in the model, which is adjusted for country/site and age-group of the woman.

The population attributable fractions (PAFs) for each category of IPV were calculated using the formula PAF = ((RRR − 1) / RRR) * Pe, where RRR is the adjusted relative risk ratio from the adjusted model and Pe is the proportion of women who had experienced that particular IPV type and who had the exposure.

Structural equation modelling (SEM) was conducted using Stata 13.0 to assess the interrelationship between variables associated with physical and/or sexual IPV in the multinomial regression model. The model outcome was a past-year IPV variable that had 4 levels drawn from the physical and sexual IPV questions: no exposure, sexual IPV, physical IPV, and physical or sexual IPV. The correlation between each hypothesized variable and the IPV variable was then tested by building variable pairs. All associations were tested by running a full-information maximum likelihood method to deal with missing values. This method was chosen over multiple imputations because it has been shown to yield superior results in structural equation modelling [22]. As a next stage, a measurement model was fitted with the variables allowed to freely correlate. To assess model fit of the observed data, we used the comparative fit index (CFI) (>0.95); Tucker-Lewis Index (TLI) (>0.9) for acceptable fit and (>0.95) as indicative of good fit [23]; and root mean square error of approximation (RMSEA) (of 0.05 or less) [24,25].

We fitted a path model using full information maximum likelihood (FIML) estimation to model all available data. The final model was built based on theory and statistically meaningful modifications using backwards elimination to exclude endogenous variables that did not mediate any path (with significance set at the *P* < 0.05 level) from the exogenous variables to IPV in order to ensure model parsimony. Before adjusting standard errors for clustering of participants in countries, model fit was very good (p(χ^2^) = 0.519, RMSEA < 0.001, CFI = 1.000, and TLI = 1.001). After adjusting for clustering, the coefficient of determination (CD) was 0.215. The model did not include any error covariances.

## Results

In total, 3,106 women aged between 18 and 49 years were interviewed in the 4 countries, among whom 2,855 (91.9%) were ever-partnered. Of the ever-partnered women, 90 (3.3%) did not respond to any of the questions related to IPV experience and were thus excluded from analysis. In total, 5,206 men were interviewed in the 4 countries, and 4,360 (83.8%) had ever been partnered. Four thousand and fifteen men completed the IPV questions, and 5,062 completed the non-partner rape questions.

### Comparison of prevalence

Comparing lifetime reports of women’s experiences and men’s reports of IPV by type from the 4 countries (Table 2) reveals that sexual IPV was quite similarly reported by men and women, except women less often disclosed lifetime sexual IPV in Cambodia (9.1% versus 21%) and China (8.3% versus 19.4%) and men reported less past-year sexual IPV in Sri Lanka. Men reported less lifetime and past-year physical IPV than women in Cambodia, but much more in China. Men reported more lifetime physical IPV than women in Bougainville, but past-year reports were similar. In Sri Lanka, the overall level of violence reported by men and women and the rates for each type were similar. In every country, women reported much more past-year emotional and financial IPV than men.

**Table 2:** Among partnered women and men, past 12-month prevalence of women’s and men’s experience of different types of violence perpetrated by their intimate partners, by country.

	Total number of partnered women/men sampled	No violence#	Sexual violence only#	Physical violence only#	Both physical and sexual violence#	Multiple emotional/economic violence#
CAMBODIA						
Women	410	43.4% (38.2%–48.8%)	3.2% (2.0%–4.9%)	16.2% (12.1%–21.3%)	5.9% (3.7%–9.4%)	31.4% (27.4%–35.6%)
Men‡	1,390	42.6% (39.6%–45.7%)	16.5% (14.6%–18.7%)	12.1% (10.2%–14.2%)	4.5% (3.6%–5.7%)	24.2% (22.0%–26.7%)
**Past year**						
Women	410	64.2% (59.2%–68.9%)	2.0% (1.0%–3.7%)	4.4% (2.3%–8.3%)	1.7% (1.0%–3.7%)	27.7% (23.4%–32.5%)
Men	1,395	87.5% (85.5%–89.3%)	3.9% (2.9%–5.2%)	2.6% (1.9%–3.5%)	0.4% (0.2%–0.8%)	5.7% (4.5%–7.1%)
CHINA						
Women	1,033	56.4% (53.1%–59.7%)	3.5% (2.5%–4.8%)	30.3% (27.4%–33.3%)	4.8% (3.6%–6.3%)	5.1% (3.8%–6.7%)
Men‡	930	44.2% (40.7%–47.8%)	6.8% (5.5%–8.4%)	32.2% (28.9%–35.6%)	12.6% (10.7%–14.8%)	4.3% (3.3%–5.6%)
**Past year**						
Women	1,033	82.6% (80.2%–84.8%)	2.3% (1.6%–3.4%)	4.9% (3.8%–6.5%)	1.0% (0.3%–1.4%)	9.4% (7.8%–11.3%)
Men	931	78.5% (76.1%–80.7%)	5.6% (4.4%–7.1%)	12.6% (10.6%–14.8%)	1.7% (1.1%–2.6%)	1.6% (1.0%–2.6%)
BOUGAINVILLE						
Women	787	25.9% (22.3%–29.9%)	16.0% (13.1%–19.4%)	11.1% (8.9%–13.7%)	40.3% (35.8%–45.0%)	6.7% (4.9%–9.2%)
Men‡	714	12.7% (9.7%–16.5%)	18.2% (15.5%–21.3%)	20.6% (17.4%–24.2%)	41.2% (36.8%–45.7%)	7.3% (5.4%–9.7%)
**Past year**						
Women	787	57.1% (52.3%–61.7%)	10.8% (8.6%–13.4%)	9.0% (7.1%–11.5%)	12.3% (9.9%–15.2%)	10.8% (8.7%–13.3%)
Men	678	60.5% (55.9%–64.8%)	15.6% (12.8%–19.0%)	12.2% (10.2%–14.6%)	6.2% (4.7%–9.2%)	5.5% (4.0%–7.3%)
SRI LANKA						
Women	535	66.2% (61.4%–70.6%)	6.9% (4.9%–9.7%)	12.2% (9.5%–15.4%)	8.4% (5.7%–12.3%)	6.4% (3.9%–10.1%)
Men‡	1,040	60.6% (57.1%–63.9%)	9.5% (7.7%–11.8%)	16.3% (14.1%–18.9%)	6.7% (5.0%–9.1%)	6.8% (5.4%–8.6%)
**Past year**						
Women	535	78.6% (73.7%–82.8%)	12.5% (8.9%–17.2%)	2.2% (1.2%–4.0%)	2.6% (1.5%–4.5%)	4.1% (2.8%–6.0%)
Men	1,011	88.5% (85.8%–90.8%)	4.9% (3.7%–6.6%)	4.3% (3.3%–5.5%)	0.9% (0.4%–1.8%)	1.4% (0.8%–2.3%)
Combined sample (women) (lifetime)	2,765	47.7% (44.9%–50.5%)	7.7% (6.5%–9.1%)	19.2% (17.5%–21.0%)	15.8% (13.5%–18.3%)	9.7% (8.2%–11.4%)
Combined sample (women) (past year)	2,765	71.8% (69.5%–74.1%)	6.7% (5.5%–8.0%)	5.5% (4.6%–6.5%)	4.5% (3.6%–5.6%)	11.5% (10.1%–13.1%)

Data are *n* or % (95% CI).

^#^ The violence categories are mutually exclusive. No violence = never experienced sexual or physical violence or multiple incidents of emotional or economic violence. Sexual violence only = experienced sexual violence with or without emotional or economic violence but did not experience physical violence. Physical violence only = experienced physical violence with or without emotional/economic violence but did not experience sexual violence. Both physical and sexual violence = experienced both physical and sexual violence with or without emotional/economic violence. Multiple emotional/economic violence only = experienced repeated incidents of emotional/economic violence but never experienced sexual or physical violence.

In Cambodia, 0.4% (95% CI 0.1%–1.73%) of women had experienced nonpartner rape in the past year, and 1.9% (95% CI 1.12%–2.70%) of men disclosed perpetration. In China, 2.3% (95% CI 1.49%–3.43%) of women had experienced nonpartner rape in the past year, and 1.7% (95% CI 0.94%–2.52%) of men disclosed perpetration. In Bougainville, 5.7% (95% CI 4.21%–7.75%) of women had experienced nonpartner rape in the past year, and 11.7% (95% CI 9.02%–14.30%) of men disclosed perpetration. In Sri Lanka, 0.5% (95% CI 0.15%–1.40%) of women had experienced nonpartner rape in the past year, and 0.8% (95% CI 0.22%–1.43%) of men disclosed perpetration.

The prevalence of past-year physical and/or sexual IPV experience increased with age (Table 3). Poverty, indicated by present food insecurity and problems finding money for an emergency, was associated with a greater risk of IPV, as was the women being the main breadwinner. Families in which the wife provided most of the money for the home were twice as likely to have food insecurity (*P* < 0.001) as those in which the husband provided, another provided, or both the husband and wife shared equally in providing.

**Table 3:** Prevalence and distribution of factors associated with women’s past-year experience of sexual or physical intimate partner violence (*N* = 2,765).

	Total *N*	*n*	Risk factor prevalence	Risk factor prevalence	Crude OR (95% CI)	aOR (95% CI)
Age	2,763					
18–24 years		450	16.8	13.9		
25–34 years		1,002	36.3	36	1.20 (0.87–1.66)	1.00 (0.66–1.50)
35–49 years		1,311	46.9	50.1	1.29 (0.93–1.78)	1.26 (0.83–1.90)
Education	2,762					
None		195	6.9	8.0		
Incomplete primary		404	13.6	19.5	1.22 (0.78–1.91)	0.97 (0.57–1.66)
Complete primary		482	16.7	21.3	1.09 (0.69–1.73)	0.99 (0.59–1.68)
Incomplete secondary		1,079	41.1	28.9	0.60 (0.38–0.95)	0.99 (0.57–1.71)
Complete secondary/higher		602	21.7	22.3	0.88 (0.55–1.42)	0.84 (0.44–1.58)
Present food insecurity	2,713	941	31.3	51.4	2.32 (1.80–3.00)	**
Resource mobilisation problems	2,717	1,519	54.1	64.8	1.56 (1.24–1.98)	**
Overall wealth score (mean [SD])	2,722		6.0 (1.5)	5.5 (1.5)	0.82 (0.76–0.88)	0.88 (0.80–0.97)
Currently married	2,763	2,432	87.4	90.9	1.43 (1.01–2.03)	2.45 (1.37–4.38)
Source of income	2,759					
Woman		293	9.7	15.3		
Her partner		1,109	40.7	37.5	0.58 (0.41–0.83)	0.83 (0.49–1.41)
Both equally		1,013	36.4	38.3	0.67 (0.49–0.92)	1.11 (0.69–1.78)
Parents/others		344	13.2	8.9	0.43 (0.27–0.69)	0.74 (0.39–1.40)
**Victimisation**						
Sexually abused as child	2,728	208	5.9	16.1	3.06 (2.23–4.21)	2.18 (1.46–3.26)
Physically abused as child	2,733	830	26.3	50.4	2.85 (2.25–3.62)	1.48 (1.11–1.97)
Emotionally abused as child	2,714	1,531	52.4	76	2.87 (2.26–3.64)	1.88 (1.41–2.51)
Witnessed abuse of mother	2,728	775	24.6	47	2.71 (2.14–3.42)	1.24 (0.96–1.60)
**Partner characteristics**						
Earning disparity	2,093					
Same		680	32.4	32.8		*
Man earns more		710	35.9	25.1	0.69 (0.52–0.92)	
Woman earns more		703	31.7	42.1	1.31 (0.98–1.76)	
Partner alcohol use	2,594					
Never		971	39.6	26.8		
Occasional		1,029	39.1	42.7	1.62 (1.24–2.11)	1.50 (1.10–2.03)
Daily or weekly		594	21.4	30.5	2.11 (1.63–2.73)	1.53 (1.12–2.08)
Partner drug use	2,735					
None		2,467	91.8	82.4		
Prior		110	3.6	6.1	1.88 (1.21–2.92)	0.85 (0.49–1.45)
Past year		158	4.6	11.5	2.78 (1.83–4.23)	1.67 (1.06–2.62)
Not confident in partner fidelity	2,702	1,014	35.1	49.5	1.81 (1.47–2.23)	1.36 (1.05–1.77)
Partner unemployed	2,717	701	22.8	40.4	2.30 (1.82–2.89)	1.18 (0.85–1.62)
Woman’s control in the relationship	2,739					
High		596	22.0	20.6		
Medium		1,786	67.1	55.7	0.89 (0.67–1.17)	0.92 (0.67–1.28)
Low		357	10.9	23.6	2.32 (1.64–3.27)	1.76 (1.15–2.69)
**Woman’s gender attitudes and relationship practices**						
Frequency of quarrelling	2,715					
Rarely		998	44.7	36.9		
Sometimes		1,143	51.1	42.5	1.01 (0.78–1.31)	1.36 (1.01–1.83)
Often		166	4.1	20.7	6.04 (4.28–8.54)	5.03 (3.17–7.99)
Cambodia‡		408	–	–	–	
Gender equity	2,763					
High		416	16	10.4		
Medium		1,632	58.8	60.5	1.58 (1.13–2.21)	0.77 (0.49–1.21)
Low		715	25.2	29.1	1.77 (1.50–2.60)	0.71 (0.41–1.23)

aOR, adjusted odds ratio.

^‡^ Item not asked in Cambodia;

* not included in the adjusted model due to high level of missing data;

** not included in the adjusted model, used ‘overall wealth score.’

### Risk factor analysis

All 3 forms of childhood abuse (sexual, physical, and emotional) and witnessing abuse of mother were more common among women with past-year physical or sexual IPV experience. Women whose partners earned more than them had a lower past-year IPV prevalence than those earning the same as their partners or women who earned more. Partner characteristics associated with women’s past-year IPV experience were the male partner’s regular alcohol use, ever or past-year drug use, lack of fidelity, and unemployment. Women who were highly controlled by their partner were more likely to have experienced past-year IPV, as were those who quarrelled more often and those holding less gender-inequitable views.

S1 Table shows the prevalence of women’s social characteristics, victimisation history, partner characteristics, and gender attitudes and relationship factors by lifetime IPV exposure category for the combined dataset (all 4 countries), with the unadjusted associations and the adjusted associations shown in S2 Table. These tables show very similar patterns of associated factors as was seen in the past-year physical or sexual IPV exposure analysis.

Table 3 shows the logistic regression models of factors associated with past-year IPV. In the past 12 months, 461/ 2,765 (16.7%) women had experienced sexual or physical (or both forms of) IPV. The risk factors shown are experiencing more poverty; having experienced abuse in childhood (sexual, physical, or emotional); having a partner who drinks alcohol, uses drugs, may be unfaithful, is unemployed, or is highly controlling; and having more frequent quarrelling in the relationship. The PAF was the largest for quarrelling often, but the second greatest PAF was for the group related to exposure to violence in childhood, followed by the PAF for the group related to the woman being controlled by her partner. The partner characteristics (substance abuse, unemployment, and infidelity) had the next highest PAFs. In the backwards/forwards elimination model, currently married women were at much higher risk.

### Structural model

**Fig 1:**
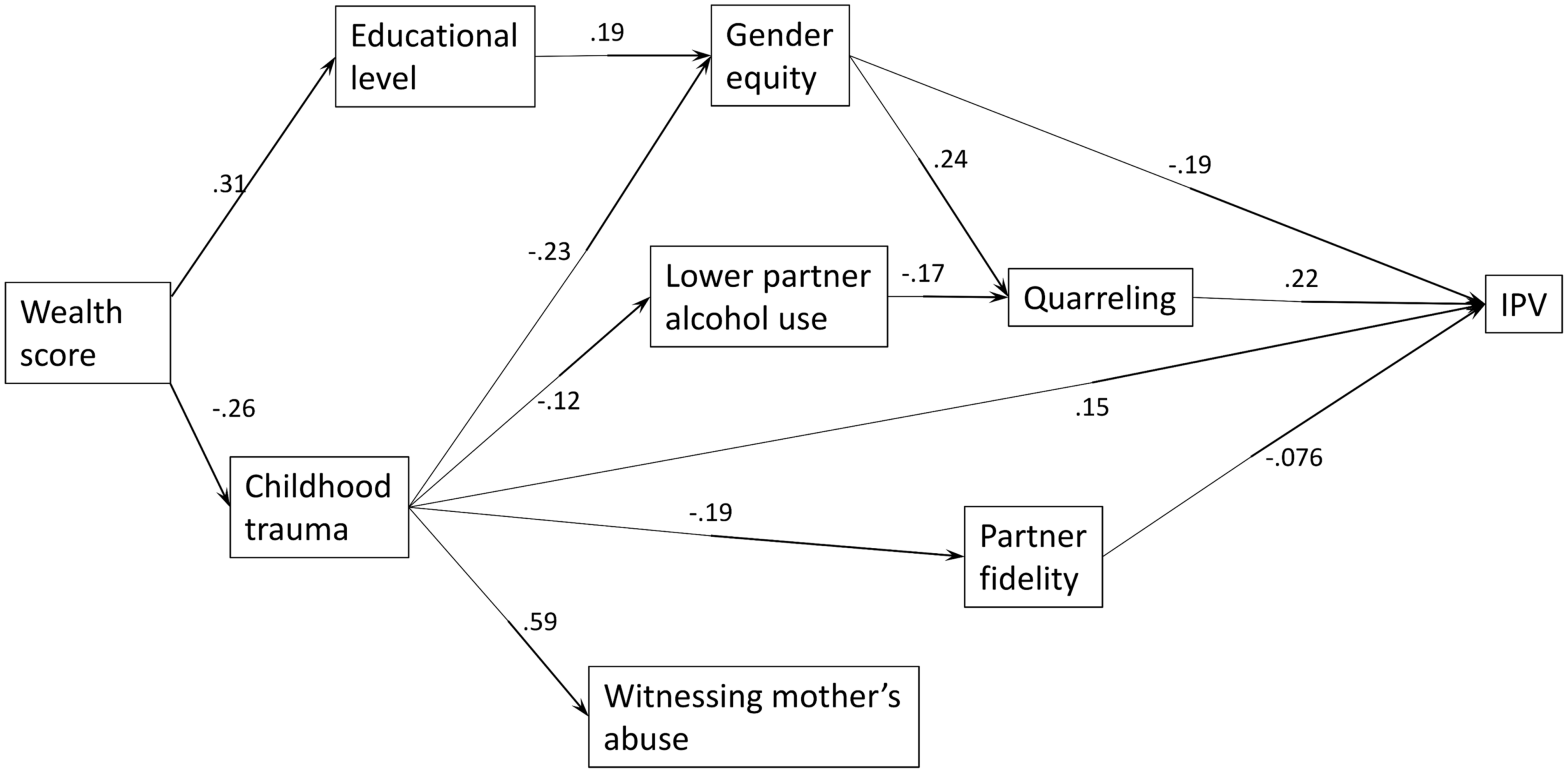
Final structural model of final factors influencing women’s experience of intimate partner violence (IPV) (standardized path coefficients [only statistically significant paths shown]).

Results for the structural equation model are presented in Fig 1 and Table 4 and follow recommended guidelines outlined by Mueller and Hancock [26]. The paths between socioeconomic status and IPV were mediated by childhood trauma exposure (i.e., poorer women had a higher trauma exposure) and increased IPV risk or by women’s educational attainment (i.e., wealthier women had been in school for longer) and having more equitable gender attitudes, which conveyed IPV protection, unless associated with more quarrelling. Childhood trauma was linked to IPV through 4 pathways. One was direct, such that childhood trauma increased the risk of IPV. One was mediated by partner alcohol use and frequency of quarrelling, such that childhood trauma reduced the chance of having a low-alcohol-using partner and thus lower quarrelling. One path was mediated by (more inequitable) attitudes to gender equity. The fourth path was mediated by partner fidelity such that risk was associated with greater confidence in him being faithful. Witnessing abuse of the woman’s mother was more common in women exposed to trauma in childhood and was included to improve model fit but did not mediate a pathway. A figure with all significant and nonsignificant paths and standard errors is presented in S1 Fig.

**Table 4:** Women's path model: Direct effects, disturbance variances, and equation-level goodness of fit.

Parameter	Standardized coefficients	SE	z	*P* > |z|	(95% confidence interval)	
Direct effects						
Wealth score → Childhood trauma	−0.257	0.058	−4.44	<0.001	−0.371	−0.144
Childhood trauma → Educational attainment	−0.056	0.045	−0.125	0.212	−0.145	0.032
Wealth score → Educational attainment	0.311	0.085	3.65	<0.001	0.144	0.478
Gender equity → Lower partner alcohol use	0.081	0.044	1.83	0.068	−0.006	0.167
Childhood trauma → Lower partner alcohol use	−0.117	0.014	−8.1	<0.001	−0.145	−0.089
Childhood trauma → Witness abuse	0.588	0.037	15.76	<0.001	0.515	0.661
Educational attainment → Partner fidelity	−0.038	0.045	−0.86	0.391	−0.126	0.049
Gender equity → Partner fidelity	0.029	0.038	0.76	0.447	−0.045	0.102
Childhood trauma → Partner fidelity	−0.194	0.038	−5.06	<0.001	−0.285	−0.119
Gender equity → Quarrelling	0.241	0.055	4.34	<.001	0.132	0.349
Lower partner alcohol use → Quarrelling	−0.174	0.063	−2.78	0.005	−0.296	−0.051
Educational attainment → Gender equity	0.187	0.071	2.63	0.009	0.047	0.326
Childhood trauma → Gender equity	−0.226	0.034	−6.61	<0.001	−0.293	−0.159
Witness abuse → Gender equity	−0.054	0.041	−1.33	0.185	−0.134	0.026
Wealth score → Gender equity	0.213	0.120	1.77	0.076	−0.022	0.448
Gender equity → IPV	−0.188	0.014	−4.53	<0.001	−0.269	−0.107
Childhood trauma → IPV	0.154	0.029	5.4	<0.001	0.098	0.210
Partner fidelity → IPV	−0.076	0.019	−4	<0.001	−0.113	−0.038
Quarrelling → IPV	0.223	0.016	13.56	<0.001	0.171	0.251
Witness abuse → IPV	0.064	0.037	1.75	0.08	−0.008	0.137
Disturbance variances	Estimate	SE			(95% confidence interval)	
Educational attainment	0.891	0.056			0.788	1.008
Gender equity	0.784	0.102			0.608	1.012
Childhood trauma	0.934	0.030			0.877	0.994
Partner fidelity	0.959	0.019			0.923	0.996
IPV	0.860	0.025			0.813	0.910
Lower partner alcohol use	0.974	0.008			0.957	0.990
Quarrelling	0.922	0.018			0.887	0.958
Witness abuse	0.655	0.044			0.574	0.746
Equation-level goodness of fit	r-squared					
Educational attainment	0.1087					
Gender equity	0.2156					
Childhood trauma	0.0662					
Partner fidelity	0.0408					
IPV	0.1397					
Lower partner alcohol use	0.0265					
Quarrelling	0.0781					
Witness abuse	0.3453					

Note: mc^2^ is the Bentler-Raykov squared multiple correlation coefficient. IPV, intimate partner violence.

## Discussion

### Comparing reports

Between a quarter and two-thirds of women in the 4 countries studied had experienced IPV, and 1.7% and 15.9% had experienced nonpartner rape. There was very great diversity in the prevalence of IPV between countries, as previously reported in Asia and the Pacific [1]. Reports by men and women show much similarity, but overall, women’s reported prevalence of lifetime physical and sexual violence experience was lower than men’s reports of perpetration, notably in sexual violence reporting. Men’s reporting of past-year nonpartner rape was much higher than women’s in Bougainville. A different pattern was seen in past-year reports that were not clearly patterned with respect to those of men, except in the area of emotional/financial abuse, for which in all countries women reported much more.

We would not necessarily expect men’s and women’s reports of nonpartner sexual violence to concur, and some women are at much higher risk than others in the population and may experience multiple rapes [27]. Although we did not have couples’ reports on partner violence, we do expect the acts/experiences of violence of men and women to be similar at a population level for past-year violence, as 75% of men had had only 1 sexual partner in the last year, and most women were married (77.7%) or cohabiting (2.9%). It is possible that women tended to minimise or forget some lifetime experiences of partner violence, but it may also be the case that higher levels of reports by men are explained by men using violence on some types of female partners more often than on their wives. Given the differences in men’s and women’s lifetime reports, we must conclude that the current global lifetime prevalence rates that are based on women’s reported experiences may underestimate the lifetime perpetration of IPV and nonpartner rape by men.

### Risk factors and drivers

We saw 4 important groups of risk factors for IPV experience. First, our results confirm that past-year IPV victimisation is more common in a context of poverty [6]. Secondly, exposure to physical, sexual, and/or emotional childhood trauma was very strongly associated with experience of all forms of IPV (past year or lifetime). This advances current research that has focused on sexual violence or on witnessing maternal abuse [5,6]. In the structural model, childhood trauma had a direct pathway to IPV experience, and it mediated several indirect paths. This helps explain why childhood trauma exposure is such an important risk factor (as shown by the PAF). The analysis of factors associated with IPV perpetration by men has also shown the importance of all forms of childhood trauma [10]. We observed also that childhood trauma exposure was associated with a more conservative position towards gender equity. It is possible that this is easier for women to adopt if they have lower self-esteem and more insecurity after trauma, as it generally is socially rewarded and normative.

Witnessing abuse of one’s mother has been found to be associated with both experience of and perpetration of IPV in many studies [17,28–35]. We confirmed this, but in the structural model, it was not as important as childhood trauma. Since previous research has often focused on witnessing abuse rather than more thoroughly measuring childhood trauma, it is possible that assumptions that there is a direct intergenerational learning process normalising IPV victimisation among women and girls are overemphasising this 1 traumatic experience, and witnessing abuse may be better interpreted as an indicator of exposure to wider childhood experiences of emotional and other trauma, all of which elevate IPV risk. The latter explanation fits better with the knowledge that witnessing abuse of one’s mother is traumatic and repulsive, which has long been an observation that fits uncomfortably with a direct learning explanation.

The third variable group consists of partner characteristics: his drinking, past-year drug use, controlling behaviour, unemployment, and fidelity. Generally, these are previously well-established risk factors, although research with men has not confirmed associations with drug use in Asia and the Pacific, except in relation to perpetration of multiple perpetrator rape [8,10]. Alcohol abuse combines a direct impact on behaviour, financial tensions, and gender-inequitable masculinity; the fidelity measure reflects the male sexual entitlement dimension of the latter [6,8,36,37]. Highly controlling behaviour is an abusive practice that is closely related to the use of physical and sexual violence [38] and is viewed by some authors as part of the concept of emotional abuse. In the structural model, male partner alcohol consumption and infidelity both mediated pathways between childhood trauma and IPV experience—in the former case, mediated by frequency of quarrelling. These partner variables may highlight the potential for enhanced prevention intervention impact if men and women are both involved in interventions to reduce violence [39]. Partner unemployment was significant on 1 of the models and would generally be interpreted as contributing to poverty in the relationship, with associated tensions, but it may also impact on self-perceived manliness, and violence may be used as a response to this [39].

The frequency of quarrelling was very strongly associated with IPV, as it was in the models of men’s perpetration in the 4 countries [10]. Although quarrelling is linked to men’s and women’s ideas about gender equity, intervention research shows that it can be reduced within relationships by training in communication skills, and this can reduce partner violence [40].

One of the most important findings of the structural model was a pathway that can be interpreted as indicating variables that build women’s resilience to violence. This linked higher wealth, higher educational attainment, and having more gender-equitable attitudes. This is very important because all of these factors are amenable to intervention, and it highlights the role of poverty reduction and interventions to enhance girls’ schooling, which may be supported for many reasons related to development and the general upliftment of women, in IPV prevention. In this study, the Gender Equitable Men (GEM) scale was used to measure women’s gender attitudes. This is a broad measure that includes attitudes towards the use of violence against women. The latter alone have been shown to be very strongly associated with risk of violence [41,42]; however, we found strong correlations with IPV in a version of the scale without the question about attitudes towards violence.

Economic empowerment has been shown to be a fruitful area of intervention with women [43], but more consistently so when combined with a gender empowerment intervention [44]. Our analysis suggests that interventions with adult women would do better to include a focus on gender empowerment and relationship dynamics in order to ensure that empowerment alone does not result in greater quarrelling and violence. Our structural model provides some indication of why interventions that impact on several variables in the resilience pathway for women (economic status and gender attitudes/relationship skills) may be much better than single-component interventions.

Reducing childhood trauma exposure is ultimately critical to reducing women’s experience of violence and is strongly related to poverty. Whilst there is much work on early interventions in childhood to reduce the experience of trauma and IPV in the next generation, it is possible that poverty reduction will have the greatest impact.

### Limitations

The study findings reflect the sampled sites; generalizability beyond this is unclear, and the combined dataset analysed here does not reflect the whole region. Since the research was cross-sectional, temporality may be questioned, but since this was recent violence, this is not likely to be a great problem. All the prevalence estimates for violence were compared with estimates weighted for the number of eligible men and women per household. The latter were not significantly different in any site, and thus, we have used unweighted estimates. The main analysis was on past-year IPV exposure, and because this is less common than lifetime exposure, the power of the analysis was inevitably impacted. However, the focus has strengthened the interpretability of the results for programming, as it is the goal of IPV prevention to reduce exposure of women at risk in the future and recent abuse is the best measure of this. A study limitation is that we do not have a comparison of men’s and women's reports from the same relationship. In accordance with WHO ethics and safety guidelines, we did not interview men and women in the same location, much less in couples. The motivation is to avoid the (to our knowledge small) possibility of retaliatory violence associated with partners learning of the interview content. This risk is not justified in cross-sectional research but prevents comparison of couples’ reports.

## Conclusions

Our findings suggest that newly emphasised past-year IPV indicators that were developed to track SDG 5 would be reasonably reliable if based on women’s interviews. Interviews with men to track past-year nonpartner rape perpetration are important. We have shown an important IPV resilience pathway. This helps us to understand why interventions that combine women’s economic empowerment and building gender-equitable attitudes (and communication skills), such as Pronyk and colleagues’ Image [43], may be more effective than those with a single-component focus. This is a very important advance in understanding as these are imminently amenable risk factors through work with populations of adult women. However, integrated approaches that reach women and men with a comprehensive set of interventions to address different risk factors would almost certainly bring the most benefit.

## Supporting information

S1 Fig Modelling showing all standardized path coefficients and standard errors (statistically significant paths shown in bold). (TIF)

S1 STROBE Checklist Strengthening the Reporting of Observational Studies in Epidemiology (STROBE) statement. Checklist of items that should be included in reports of cross-sectional studies.
(DOC)

S1 Table Prevalence of factors associated with lifetime women’s experience of intimate partner violence (IPV), by type of violence. (DOCX)

S2 Table Crude relative risk ratios (RRRs) and adjusted relative risk ratios (aRRRs) of factors associated with lifetime women’s experience of intimate partner violence (IPV), by type of violence. (DOCX)

## Abbreviations

aOR: adjusted odds ratio
APDA: audio-enhanced personal digital assistant
aRRR: adjusted relative risk ratio
GEM: Gender Equitable Men
IPV: intimate partner violence
PAF: population attributable fraction
PNG: Papua New Guinea
RRR: relative risk ratio
SDG: Sustainable Development Goal
STROBE: Strengthening the Reporting of Observational Studies in Epidemiology
UNMCS: UN Multi-country Study on Men and Violence
VAWG: violence against women and girls
