# Supplementary figures and images for: Women’s and men’s reports of past-year prevalence of intimate partner violence and rape and women’s risk factors for intimate partner violence: A multicountry cross-sectional study in Asia and the Pacific
Source: PLoS Med. 2017 Sep 5;14(9):e1002381. doi: 10.1371/journal.pmed.1002381 (PMC5584751; doi:10.1371/journal.pmed.1002381)

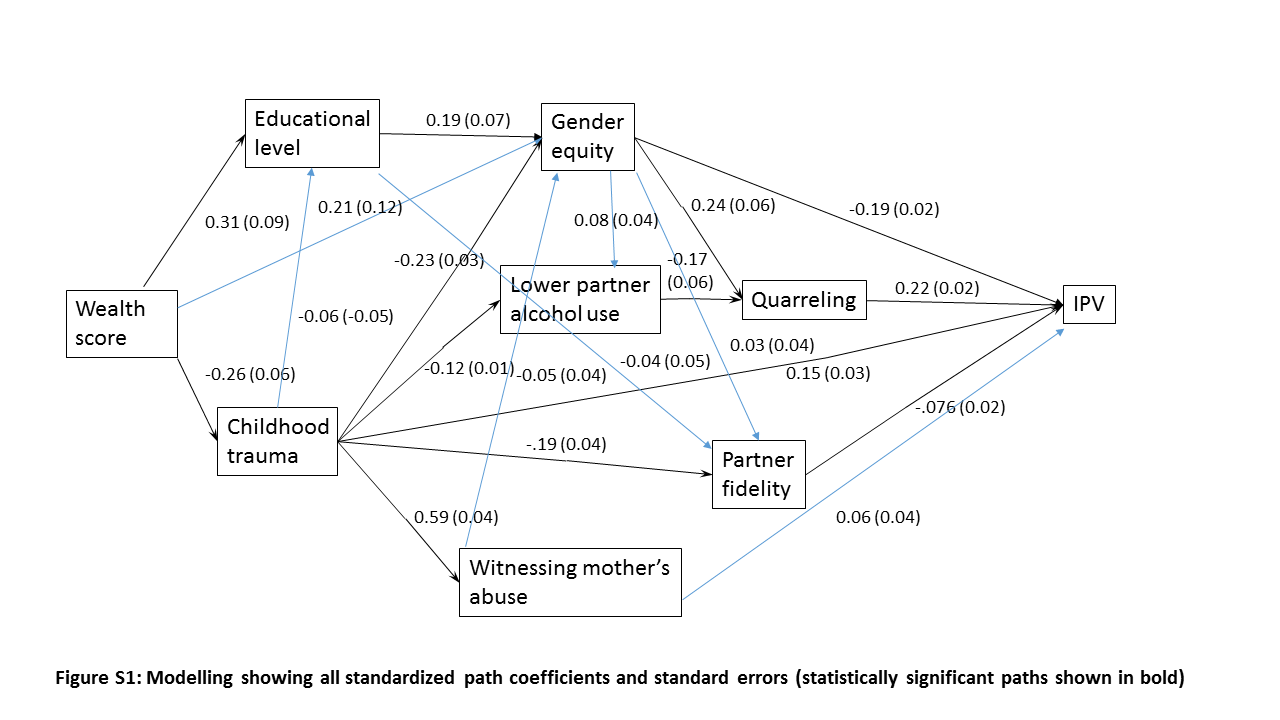

Supplement: S1 Fig — (TIF) [file pmed.1002381.s001.tif]
