# Supplementary material for: Women’s and men’s reports of past-year prevalence of intimate partner violence and rape and women’s risk factors for intimate partner violence: A multicountry cross-sectional study in Asia and the Pacific
Source: PLoS Med. 2017 Sep 5;14(9):e1002381. doi: 10.1371/journal.pmed.1002381 (PMC5584751; doi:10.1371/journal.pmed.1002381)
Supplement: S1 Table — (DOCX) [file pmed.1002381.s003.docx]

**S1 Table: Prevalence of factors associated with lifetime women’s experience of intimate partner violence, by type of violence.**

|  | Overall N | No Violence | Sexual violence only | Physical Violence only | Physical and Sexual violence | Multiple emotional/  Economic violence |
| --- | --- | --- | --- | --- | --- | --- |
| Age |  |  |  |  |  |  |
| 18-24yrs | 449 | 19.2% | 15.1% | 13.6% | 12% | 15% |
| 25-34yrs | 1001 | 34.8% | 37.3% | 33.8% | 37.9% | 44.9% |
| 35-49yrs | 1310 | 46% | 47.6% | 52.6% | 50.1% | 40.1% |
| Education |  |  |  |  |  |  |
| None | 194 | 5.8% | 6.1% | 5.7% | 8% | 12% |
| Incomplete Primary | 404 | 7.6% | 17.5% | 7.7% | 21.6% | 17.2% |
| Completed Primary | 482 | 25.6% | 25.5% | 29.2% | 25.5% | 27.7% |
| Incomplete Sec | 1078 | 39.1% | 27.8% | 37.9% | 18.4% | 26.2% |
| Complete Sec/higher | 601 | 20% | 23.1% | 19.5% | 26.4% | 16.9% |
|  |  |  |  |  |  |  |
| Present food insecurity | 2711 | 24.2% | 46.9% | 31.8% | 56.8% | 45.9% |
| Resource mobilisation problems | 2715 | 52.6% | 62.3% | 50.6% | 61.9% | 67.7% |
| Overall wealth score | 2722 | 6.1(1.4)‡ | 5.6(1.4)‡ | 6.0(1.5)‡ | 5.5(1.5)‡ | 5.4(1.5)‡ |
| Married as child (<18) | 2758 | 3.3% | 8.1% | 3.% | 11.5% | 8.2% |
| Currently married | 2762 | 86.3% | 84.4% | 91.3% | 88.7% | 91.8% |
| Source of Income |  |  |  |  |  |  |
| Woman | 293 | 6.9% | 13.2% | 11.7% | 19.8% | 9.7% |
| Her partner | 1109 | 44.4% | 36.3% | 35.2% | 31.3% | 9.7% |
| Both equally | 1011 | 35.1% | 35.4% | 42.5% | 39.6% | 29.2% |
| Parents/others | 343 | 13.5% | 15.1% | 10.6% | 9.2% | 13.9% |
| **Victimisation** |  |  |  |  |  |  |
| Sexually abused as child | 2726 | 3.5% | 8.1% | 9.1% | 18% | 7.5% |
| Physically abused as child | 2731 | 18.1% | 36% | 31.3% | 58% | 39% |
| Emotionally abused as child | 2713 | 41.5% | 67.3% | 61.8% | 82.4% | 67.3% |
| Witnessed abuse of mother | 2726 | 17.1% | 36% | 31.7% | 56.6% | 25.5% |
| **Partner Characteristics** |  |  |  |  |  |  |
| Partner alcohol use |  |  |  |  |  |  |
| Never | 970 | 46.8% | 36.3% | 31.3% | 24% | 26.5% |
| Less often | 1029 | 36.6% | 43.3% | 42.2% | 41.9% | 43.8% |
| Daily or weekly | 594 | 16.6% | 21.4% | 26.5% | 34.1% | 29.6% |
| Partner drug use |  |  |  |  |  |  |
| None | 2485 | 93.7% | 86.3% | 91.3% | 79.1% | 92.1% |
| Prior | 111 | 2.9% | 5.7% | 3.4% | 8.5% | 2.3% |
| Past year | 158 | 3.4% | 8.0% | 5.3% | 12.4% | 5.6% |
| Not Confident in Partner fidelity | 2700 | 26.3% | 38% | 48.5% | 56.5% | 38.3% |
| Partner Unemployed | 2716 | 16.1% | 44.8%% | 24.9% | 47.8% | 16.9% |
| Women’s control in relation ship |  |  |  |  |  |  |
| High | 596 | 20.4% | 23.1% | 20.6% | 20.9% | 30.7% |
| Medium | 1785 | 70.2% | 61.3% | 67.6% | 54.0% | 57.3% |
| Low | 358 | 9.4% | 15.6% | 11.7% | 25.1% | 12.0% |
| **Gender attitudes & Relationship practices**  Frequency of quarrelling* |  |  |  |  |  |  |
| Rarely | 998 | 51.6% | 61.1% | 21.4% | 39.4% | 35.5% |
| Sometimes | 1141 | 46.8% | 35.9% | 68.4% | 38.9% | 60.1% |
| Often | 166 | 1.6% | 3.0% | 10.3% | 21.7% | 4.3% |
| Women’s views on gender equity |  |  |  |  |  |  |
| High | 414 | 16.9% | 8.8% | 22.8% | 7.1% | 5.6% |
| Medium | 1631 | 59.4% | 56.1% | 57.0% | 60.5% | 53.2% |
| Low | 717 | 23.8% | 35.1% | 20.2% | 32.4% | 41.2% |
|  |  |  |  |  |  |  |
| *excludes Cambodia where the question was not asked ‡ : mean score(standard deviation) | | | | | | |
|  | | | | | | |
