# Supplementary material for: Women’s and men’s reports of past-year prevalence of intimate partner violence and rape and women’s risk factors for intimate partner violence: A multicountry cross-sectional study in Asia and the Pacific
Source: PLoS Med. 2017 Sep 5;14(9):e1002381. doi: 10.1371/journal.pmed.1002381 (PMC5584751; doi:10.1371/journal.pmed.1002381)
Supplement: S2 Table — (DOCX) [file pmed.1002381.s004.docx]

**S2 Table: Crude (RRR) and adjusted relative risk ratios (aRRR) of factors associated with lifetime women’s experience of intimate partner violence, by type of violence.**

|  | Sexual violence only | | Physical Violence only | | Physical and Sexual violence | | Multiple emotional/  Economic violence | |
| --- | --- | --- | --- | --- | --- | --- | --- | --- |
|  | Crude RRR  (CI 95%) | aRRR (CI 95%) | Crude RRR  (CI 95%) | aRRR (CI 95%) | Crude RRR  (CI 95%) | aRRR (CI 95%) | Crude RRR  (CI 95%) | aRRR (CI 95%) |
| **Social characteristics** |  |  |  |  |  |  |  |  |
| Age |  |  |  |  |  |  |  |  |
| 18-24yrs | … |  | … |  |  |  |  |  |
| 25-34yrs | 1.36(0.82-2.25) | 1.84(1.03-3.27) | 1.37(0.96-1.96) | 1.30(0.83-2.04) | 1.75(1.19-2.58) | 2.27(1.31-3.95) | 1.66(1.08-2.54) | 1.81(1.14-2.89) |
| 35-49yrs | 1.31(0.82-2.11) | 2.03(1.17-3.50) | 1.62(1.17-2.24) | 1.55(0.99-2.42) | 1.75(1.19-2.59) | 2.92(1.63-5.21) | 1.12(0.75-1.68) | 1.30(0.85-2.00) |
| Education |  |  |  |  |  |  |  |  |
| None | … |  | … |  | … |  |  |  |
| Incomplete Primary | 1.54(0.72-3.28) | 0.98(0.42-2.25) | 0.99(0.60-1.62) | 1.06(0.61-1.85) | 1.52(1.01-2.28) | 1.07(0.58-1.98) | 0.79(0.45-1.37) | 1.33(0.72-2.47) |
| Completed Primary | 1.43(0.68-3.02) | 1.13(0.50-2.56) | 1.17(0.71-1.91) | 1.16(0.67-1.99) | 1.20(0.78-1.87) | 1.24(0.69-2.23) | 0.91(0.53-1.54) | 1.96(1.05-3.68) |
| Incomplete Sec | 0.59(0.29-1.22) | 1.11(0.52-2.53) | 1.04(0.65-1.67) | 1.10(0.65-1.88) | 0.33(0.21-0.53) | 1.10(0.57-2.13) | 0.31(0.19-0.52) | 1.36(0.71-2.63) |
| Complete Sec/higher | 0.98(0.47-2.06) | 0.95(0.42-2.19) | 0.90(0.55-1.47) | 0.89(0.50-1.60) | 0.92(0.60-1.41) | 1.11(0.57-2.18) | 0.39(0.23-0.68) | 1.52(0.73-3.14) |
|  |  |  |  |  |  |  |  |  |
| Present food insecurity | 2.75(1.96-3.88) | ** | 1.46(1.16-1.83) | ** | 4.12(3.24-5.24) | ** | 2.65(1.99-3.53) | ** |
| Resource mobilisation problems | 1.49(1.04-2.13) | ** | 0.92(0.74-1.15) | ** | 1.47(1.15-1.88) | ** | 1.89(1.37-2.60) | ** |
| Overall wealth score | 0.81(0.73-0.89) | 0.82(0.72-0.93) | 0.98(0.90-1.06) | 0.96(0.87-1.07) | 0.75(0.69-0.81) | 0.82(0.73-0.92) | 0.73(0.66-0.81) | 0.99(0.87-1.13) |
| Married as child (<18) | 2.53(1.44-4.46) | 0.94(0.47-1.87) | 0.90(0.52-1.56) | 0.83(0.44-1.55) | 3.75(2.43-5.80) | 1.42(0.70-2.89) | 2.59(1.61-4.17) | 1.41(0.83-2.40) |
| Currently married | 0.86(0.57-1.30) | 2.76(1.36-5.60) | 1.67(1.17-2.37) | 2.83(1.46-5.52) | 1.25(0.81-1.92) | 5.36(2.58-11.1) | 1.76(1.03-3.03) | 3.38(1.77-6.48) |
| Source of Income |  |  |  |  |  |  |  |  |
| Woman | … |  | … |  | … |  |  |  |
| Her partner | 0.43(0.27-0.68) | 0.63(0.33-1.20) | 0.47(0.31-0.69) | 0.72(0.43-1.20) | 0.25(0.16-0.37) | 0.61(0.32-1.16) | 0.76(0.47-1.21) | 0.67(0.38-1.18) |
| Both equally | 0.53(0.32-0.86) | 0.83(0.44-1.590 | 0.71(0.50-1.03) | 0.74(0.44-1.27) | 0.39(0.27-0.57) | 0.94(0.50-1.75) | 0.59(0.37-0.94) | 0.72(0.41-1.27) |
| Parents/others | 0.58(0.31-1.09) | 1.16(0.56-2.37) | 0.46(0.29-0.73) | 0.93(0.53-1.64) | 0.24(0.15-0.38) | 0.60(0.30-1.20) | 0.73(0.40-1.31) | 1.24(0.64-2.43) |
| **Victimisation** |  |  |  |  |  |  |  |  |
| Sexually abused as child | 2.39(1.36-4.21) | 1.80(0.85-3.80) | 2.70(1.77-4.15) | 1.83(1.08-3.12) | 5.98(4.00-8.93) | 3.29(1.85-5.86) | 2.20(1.29-3.75) | 2.64(1.45-4.78) |
| Physically abused as child | 2.55(1.80-3.61) | 1.25(0.82-1.90) | 2.06(1.66-2.56) | 1.76(1.32-2.35) | 6.24(4.64-8.39) | 2.22(1.50-3.26) | 2.89(2.18-3.83) | 1.20(0.87-1.67) |
| Emotionally abused as child | 2.90(2.11-4.00) | 2.07(1.37-3.11) | 2.28(1.86-2.81) | 1.67(1.30-2.14) | 6.62(4.96-8.85) | 2.39(1.71-3.36) | 2.90(2.23-3.77) | 1.90(1.42-2.54) |
| Witnessed abuse of mother | 2.74(1.95-3.82) | 1.27(0.84-1.91) | 2.25(1.82-2.80) | 1.62(1.22-2.14) | 6.33(4.88-8.21) | 2.03(1.44-2.87) | 1.66(1.18-2.33) | 1.07(0.74-1.54) |
| **Partner Characteristics** |  |  |  |  |  |  |  |  |
| Partner alcohol use |  |  |  |  |  |  |  |  |
| Never | --- |  | --- |  | --- |  | --- |  |
| Less often | 1.49(1.05-2.11) | 1.33(0.90-1.97) | 1.72(1.35-2.21) | 1.50(1.14-1.98) | 2.23(1.67-2.99) | 1.83(1.27-2.64) | 2.11(1.46-3.05) | 1.19(0.80-1.77) |
| Daily or weekly | 1.66(1.08-2.54) | 1.66(1.05-2.62) | 2.38(1.78-3.19) | 1.94(1.38-2.71) | 4.00(2.93-5.45) | 3.51(2.36-5.23) | 3.15(2.17-4.55) | 1.86(1.25-2.76) |
| Partner drug use |  |  |  |  |  |  |  |  |
| None |  |  |  |  |  |  |  |  |
| Prior | 2.15(1.04-4.56) | 1.31(0.53-3.22) | 1.23(0.67-2.26) | 1.24(0.51-3.02) | 3.53(2.15-5.78) | 1.60(0.68-3.76) | 0.80(0.33-1.92) | 0.60(0.19-1.92) |
| Past year | 2.56(1.32-4.95) | 2.14(1.00-4.57) | 1.61(0.88-2.93) | 2.33(1.19-4.55) | 4.33(2.55-7.36) | 3.88(2.11-7.15) | 1.68(0.93-3.04) | 1.72(0.92-3.20) |
| Not Confident in Partner fidelity | 1.72(1.23-2.40) | 1.54(1.04-2.26) | 2.64(2.09-3.33) | 1.97(1.52-2.54) | 3.63(2.72-4.86) | 2.51(1.74-3.63) | 1.74(1.32-2.29) | 1.53(1.13-2.05) |
| Partner Unemployed | 4.24(3.00-5.98) | 1.87(1.18-2.97) | 1.73(1.31-2.28) | 1.61(1.14-2.26) | 4.78(3.70-6.19) | 1.24(0.87-1.78) | 1.59(1.11-2.27) | 1.33(0.89-2.00) |
| Women’s control in relation ship |  |  |  |  |  |  |  |  |
| High | --- |  | --- |  | --- |  | --- |  |
| Medium | 0.77(0.52-1.13) | 0.87(0.54-1.41) | 0.95(0.71-1.28) | 0.82(0.59-1.15) | 0.75(0.56-1.02) | 0.99(0.66-1.50) | 0.54(0.54-0.73) | 1.07(0.73-1.56) |
| Low | 1.47(0.88-2.47) | 1.61(0.89-2.90) | 1.25(0.84-1.84) | 1.20(0.75-1.93) | 2.62(1.78-3.87) | 2.34(1.46-3.74) | 0.85(0.51-1.42) | 1.00(0.59-1.72) |
| **Gender attitudes & Relationship practices**  Frequency of quarrelling* |  |  |  |  |  |  |  |  |
| Rarely | ------ |  | ------ |  | ------- |  | ------ |  |
| Sometimes | 0.64(0.46-0.92) | 1.38(0.90-2.09) | 3.5(2.69-4.59) | 3.36(2.44-4.61) | 1.09(0.81-1.47) | 2.82(1.93-4.10) | 1.87(1.25-2.80) | 3.24(2.06-5.11) |
| Often | 1.56(0.60-4.11) | 2.40(0.92-6.27) | 15.1(8.23-27.8) | 11.2(6.06-20.6) | 17.3(9.98-30.1) | 22.8(12.0-43.7) | 3.86(1.42-10.5) | 4.26(1.39-13.1) |
| Women’s views on gender equity |  |  |  |  |  |  |  |  |
| High | --- |  | --- |  | --- |  | --- |  |
| Medium | 1.61(1.03-2.52) | 0.58(0.34-0.99) | 0.71(0.55-0.92) | 0.70(0.51-0.97) | 2.41(1.53-3.78) | 0.70)0.38-1.28) | 2.69(1.58-4.57) | 1.83(1.00-3.33) |
| Low | 1.28(0.76-2.16) | 0.41(0.22-0.77) | 0.63(0.55-0.88) | 0.75(0.48-1.18) | 3.24(1.95-5.36) | 0.71(0.37-1.38) | 5.22(2.90-9.38) | 2.10(1.07-4.13) |
|  |  |  |  |  |  |  |  |  |
| RRR= relative risk ratio. *excludes Cambodia where the question was not asked ** : not included in the adjusted model ,used ‘overall wealth score” | | | | | | | | |
|  | | | | | | | | |
